# Treatment of Abscess

**Published:** 1897-12

**Authors:** 

**Affiliations:** Flint, Mich.


ARTICLE IV.

### TREATMENT OF ABSCESS.

BY DR. MONROE, FLINT, MICH.

It is not so many months ago that I have ceased to remember with pleasure the last dental convention I attended.

I recall to mind with a great deal of satisfaction the benefits which I received, in the way of personal experience, related by members of that convention.

From the narration of more than one of these experience, I received suggestions which are of great benefit to me in my every-day practice.

It has been my fortune to have some experience in a certain line of work, which is attracting no little attention at present in our dental journals. While I cannot hope that I am telling you anything new, I must at least hope to succeed in interesting you to a limited extent.

Two years ago a gentleman about 45 years of age came into my office and said: "I have some teeth that trouble me. They have been troubling me for over a year. I have been in two offices during that time and they had started to treat them, but it hurt so badly that I have never went back to either of them for a second treatment. I don't want to be hurt; I would prefer the toothache rather than to suffer pain in a dental chair."

Most of his posterior teeth had been extracted under an anæsthetic, and he was depending largely on his anterior ones for mastication. The troublesome tooth was the left cuspid superior, which was slightly raised in its socket, and a little loose. The lateral and central incisors next to it were also devitalized.

Knowing that I would probably not be able to get him in the chair for a second treatment, I determined to try to make it a clean operation at one sitting.

I opened all three teeth through their root-canals, wiped out all the pus I could press out through the canal of the cuspid, washed it out carefully with warm, carbolized water, forced carbolic acid and iodine, equal parts, into the pus cavity, and filled it with chloro-percha and gutta-percha and gutta-percha points. I then cleaned out the canals of the central and lateral, sterilized them, and filled them in the same way. I told him that they would probably trouble him for a day or so, but after that they might never bother him.

I met him on the street in a few days, and he said that the night after the treatment he slept little or none at all. The gum had swollen and burned, as he explained it, but they were then allright. In about two months from that time I put in permanent fillings and they have not troubled him since. I had occasion to treat the lingual root of a superior right second molar, which would have been almost impossible to open through the palatal side, in the same manner as the case above mentioned, with the same results, a little more than a year ago. Yet, on account of the trouble following, I have never followed this for a practice, and, in fact, have extracted molars in preference.

Prior to and since that time I have treated similarly affected teeth by cleaning out the root-canals, through the apex of the root, by using Gates' glidden drills, if necessary, or even a 50 per cent. solution of sulphuric acid. Then, after injecting about a 1½ per cent. solution of cocaine, to a mixture of tincture of iodine, carbolic acid, sulph. morphia, atropine sulph. (pot. iod.), glycerine and water into the gum, over the apex of the root, which renders the gum entirely painless, lance the gum in the form of a cross, and with a large, round, sharp bur drill through the process, completing this work with a sharp fissure bur, with which it is an easier matter to cut off the end of the root or drill out any diseased bone in the pus-cavity. The small particles of process and tooth are now washed out, first with warm carbolized water, then with peroxide of hydrogen, followed by carbolic acid and iodine, equal parts, forced through the canal and out through the fistula, leaving a thread of cotton saturated in carbolic acid and iodine in the canal, with another similar one in the fistula. The after-treatment consisted in simply washing out the cavities with perovide of hydrogen, unless the pus continued to form, when the first operation would be repeated. Sometimes a cure would be brought about after the first treatment, and again, after two or three months' treatment, the tooth would have to be extracted.

At the time of our last convention in Grand Rapids, I had a right superior central incisor that I had been treating in this way for about two months, with no hope of success. I consulted several of the doctors present in regard to it and received as many different replies. As they all had some failures, I concluded that I would have to extract it. But before doing so I cleaned it out thoroughly, sterilized it well through the canal and fistula and filled the canal permanently, after I had thoroughly dried it with tannic acid and cotton. The patient remarked that the tooth felt better than it had at any previous treatment. Every few days I would wash out the fistula, which remained open of itself, due to the purulent discharge that had no other avenue of escape, with warm, carbolized water, and inject a few drops of tincture of iodine into the pus-cavity. In a short time the opening healed up and has since caused no trouble.

Since that time I have been filling the root-canals of abscessed teeth at the first sitting, after the opening has been made through the process and the pus-cavity thoroughly burred out and cleansed. I fail to see of what benefit it is to leave the root and canal unfilled. To my mind, it would be as easy to treat a chronic case of appendicitis through the oral cavity as to treat a chronic abscessed tooth through the root canal. When we thoroughly clean out a root-canal, open well through the apex to the fistula, we have, in my opinion, done all we can through that channel. It is better to fill it at once, and the fistula will allow any pus to escape that might otherwise be enclosed. Should any pus form afterward, it is evident that we have a diseased tooth to drill out, which can be reached only through the fistulous opening. That we have a diseased tooth and not process is proven by the fact that when we extract an abscessed tooth, the cavity left generally heals up in a few days, causing no further trouble. After cutting off the end of the root, we have the diseased portion extracted, and the cavity, represented by the fistula, equals the cavity made where the tooth was extracted.

This is the way I, with my limited experience, view the matter, and if my views are mistaken or exagerated, my heartfelt thanks will be given to him who shows me wherein I have erred.

### DISCUSSION.

Dr. Williams.—I would like to know if it would not be of more benefit to leave the canal open instead of filling it permanently. I fill it with cotton, which is not so difficult to extract as gutta percha.

Dr. Monroe.—I do not think it necessary to remove the filling. The difficulty would be at the root or in the process. I think a tooth can be over-treated. The point I wished to make was that of filling the tooth at the first sitting.

Dr. House.—I would like to ask Dr. Monroe if that pertains to teeth where the pulp has become devitalized, where it has decomposed, where the pulp is dead, or where it is absorbed entirely. We often find teeth where the pulp is devitalized and the flow of pus difficult to arrest. Also cases where the pus is absorbed and we have a dry condition to act upon.

Dr. Monroe.—I had a case yesterday of that kind, which I am to extract. I should treat it at first, for the reason that it is sore. It is a left lateral incisor and there is a hard lump under the eye. I believe in that case it would be better to wait. It would be easier for the patient. But in the case of the tooth being either dry, or pus coming through the root-canal, clean it out thoroughly and sterilize; if blood or serum continues through the canal, use tannin and cotton. Sometimes it will take an hour and a half to dry and fill it. In the case to which I referred, the patient said the tooth felt all right. The fistula is still open.

Dr. Warboys.—I would like to ask Dr Monroe if he has ever had a recurrence of an abscess after that kind of treatment after he considered it entirely healed up?

Dr. Monroe.—More often than otherwise. Even after cutting off a portion of the root.

Dr. Young.—I have always considered it best to fill the tooth before cutting off the root, because after cutting it off you must have a larger opening, and it is more difficult to fill on account of pushing the filling farther than you wish it to go. I think a permanent filling helps to force the pus out.

Dr. Morgan.—I have claimed for a long time that it was impossible to make a lower molar healthy after once diseased. I have met with better success if, after opening into the root, I had used a hot instrument at once before washing. I have always been very careful not to drill through the end of the root. If an upper front tooth, usually the opening at the end of the root is large enough to force the medicine through without drilling.

Dr. Monroe.—I have found oftentimes that by drilling through the tooth, getting a circuit through the fistulous opening and root-canal, and after drying thoroughly and filling, that the fistula would heal itself.—*Dental Register.*

